# Obesity and *Staphylococcus aureus* Nasal Colonization among Women and Men in a General Population

**DOI:** 10.1371/journal.pone.0063716

**Published:** 2013-05-07

**Authors:** Karina Olsen, Kjersti Danielsen, Tom Wilsgaard, Maria Sangvik, Johanna U. E. Sollid, Inger Thune, Anne E. Eggen, Gunnar S. Simonsen, Anne-Sofie Furberg

**Affiliations:** 1 Department of Microbiology and Infection Control, University Hospital of North Norway, Tromsø, Norway; 2 Department of Community Medicine, Faculty of Health Sciences, University of Tromsø, Tromsø, Norway; 3 Department of Dermatology, University Hospital of North Norway, Tromsø, Norway; 4 Research Group for Host-Microbe Interactions, Department of Medical Biology, Faculty of Health Sciences, University of Tromsø, Tromsø, Norway; 5 Department of Oncology, Oslo University Hospital, Ullevål, Oslo, Norway; Oxford University, Viet Nam

## Abstract

**Background:**

Obesity and diabetes mellitus (DM) have been linked to increased risk of infections, and *Staphylococcus aureus* nasal colonization is a major risk factor for developing infections with the microbe. We therefore sought to find whether body mass index (BMI) and waist circumference (WC) could be associated with *S. aureus* colonization independent of DM.

**Methodology:**

*S. aureus* colonization was assessed by nasal swab cultures among 2,169 women and 1,709 men, aged 30–87 years, in the population-based Tromsø Staph and Skin Study in 2007–08. Height (cm), weight (kg), WC (cm), and glycated haemoglobin (HbA1c,%) were measured. Multivariable logistic regression analyses including information on DM, HbA1c, hormonal contraceptive use and other potential confounders were used.

**Results:**

In the female population, each 2.5 kg/m^2^ increase in BMI was associated with a 7% higher odds of *S. aureus* nasal colonization (*P = *0.01). When comparing obese and lean women aged 30–43 years, we observed that BMI ≥32.5 versus <22.5 kg/m^2^ and WC ≥101 versus <80 cm was associated with a 2.60 and 2.12 times higher odds of *S. aureus* colonization, respectively (95% confidence intervals 1.35–4.98 and 1.17–3.85). Among men, high WC was also associated with *S. aureus* nasal colonization. The associations did not change significantly when the analysis was restricted to participants without signs of pre-diabetes (HbA1c <6.0%) among women and men, and to non-users of hormonal contraceptives among women.

**Conclusion:**

Our results support that obesity is a possible determinant for *S. aureus* nasal colonization independent of DM, in particular for premenopausal women. The role of obesity at different ages and by sex should be addressed in future prospective studies of *S. aureus* colonization.

## Introduction

Overweight and obesity are established risk factors for major chronic diseases, but their effects on susceptibility to infections are not yet fully understood [1], [2]. Previous studies have observed that obese patients are more likely to develop community-acquired pneumonia and wound infections, as well as nosocomial sepsis, bacteremia, surgical site infections and catheter-related infections [1], [3]–[6]. *Staphylococcus aureus* is a frequent causative agent in several of these infections.

Persistent nasal colonization of *S. aureus* which occurs in about 20–30% of healthy adults is a major risk factor for infections with the bacterium [7]–[11]. Thus, modification of factors predisposing to colonization may contribute substantially in reducing the *S. aureus* disease burden [12]. Interestingly, obesity was associated with *S. aureus* nasal colonization among both men and women in the US National Health and Nutrition Examination Survey (NHANES) 2001–04 [13]. Obesity has also been identified as a risk factor for preoperative *S. aureus* nasal colonization among surgical patients [14]. Elevated serum glucose concentration and type 2 diabetes are often linked to obesity [15] and have been associated with *S. aureus* nasal colonization [16], [17], suggesting that altered glucose metabolism may mediate obesity-related effects on immune responses [1], [16], [18]. However, studies in humans and animals suggest that adiposity in itself may also cause impaired immune responses through inflammatory and sex-steroid signalling pathways [2], [19]–[25].

Thus, the role of obesity as an independent risk factor for *S. aureus* colonization in a general population is not clarified and studies are limited. As obesity has become endemic worldwide, even small increases in risk may have major impact on the overall *S. aureus* disease burden in a population. Therefore, the aim of this study was to investigate associations between excess body weight and abdominal adiposity and *S. aureus* nasal colonization using analyses adjusted for pre-diabetes and diabetes mellitus (DM) among a large population-based sample of women and men.

## Materials and Methods

### Ethics

The study was approved by the Regional Committee of Medical and Health Research Ethics, North Norway (REK Nord No.: Ref 200605174–12/IAY/400) and complied with the Declaration of Helsinki. All participants enrolled in the study signed an informed consent form before participation.

### Population and study design

The participants in the Tromsø Staph and Skin Study (TSSS) were recruited from a population-based study, the sixth Tromsø Study (Tromsø 6), carried out from October 2007 to December 2008 with 12,984 participants and an attendance rate of 65.7%. The Tromsø Study is a longitudinal multipurpose study focusing on lifestyle related diseases [26], [27]. Nasal swab cultures were collected in a random sample of 4,026 participants aged 30–87 years, during October 2007 to June 2008, estimated to give sufficient power for subgroup analysis of host-microbe relationships in the TSSS [7], [28]. Among these, 129 nasal swab cultures were considered invalid due to either use of antibiotics within the last 24 hours (*n* = 27) or no bacterial growth in cultures (*n = *102). Pregnant women (*n* = 15) and participants with missing height and/or weight data (*n* = 4) were excluded, leaving 3,878 participants for analysis of BMI. In addition, 103 participants with missing WC data were excluded, leaving 3,775 participants for analysis of WC.

Interviews, clinical examinations, nasal swab cultures, and blood samples were performed according to standardized procedures by trained health personnel at the screening centre. Two self-administered structured questionnaires covered a broad range of issues related to socioeconomic status, lifestyle, and health and disease including DM [27].

### Assessment of *S. aureus* nasal colonization

Both vestibuli nasi were sampled by the same NaCl-moistened, sterile rayon-tipped swab and placed in Amies charcoal transport medium (Copan, Brescia, Italy). Within 3 days, all specimens were cultured on blood agar (Oxoid, Cambridge, UK), chromId™ *S. aureus* and chromId™ MRSA agars (bioMérieux, Marcy I′Etoile, France), and incubated for 42–48 hours at 37°C. If positive (green) colonies were found on the chromId plates, the most dominating colony was selected and confirmed as *S. aureus* by the Staphaurex Plus (Remel, Lenexa, KS, USA) agglutination test. All *S. aureus* isolates were frozen at –70°C in glycerol-containing liquid media. No methicillin-resistant *S. aureus* (MRSA) was detected [7], [29]. The *S. aureus* colonization state was determined by a single nasal swab culture taken at the screening. This decision was based on the evaluation of the agreement between culturing results in a sub-cohort of the TSSS including 2,868 participants who made a second visit to the screening centre and had a second nasal swab culture taken after a median time of 28 days. In 90% of these participants the interval was ≥12 days, and only 113 of the 2,868 participants (3.9%) were misclassified as colonized from the culturing results of the first nasal swab (i.e. first swab culture positive and second swab culture negative). Among those with two positive swab cultures (*n* = 727), 669 participants (92%) had the same *spa* type in both samples.

### Measurement of body mass index and waist circumference

Body height in centimetres (cm) and weight in kilograms (kg) were measured to the nearest 0.1 unit wearing light clothing and no shoes. BMI was calculated as weight divided by height squared (kg/m^2^). WC was measured at the umbilical line to the nearest cm [26], [27]. The World Health Organization (WHO) defines BMI ≥30.0 kg/m^2^ as obesity [30] and WC values >88 cm and >102 cm in women and men, respectively, as high risk abdominal obesity [31].

### Measurements of other covariates

Smoking status was coded as ‘Current daily smoking’ (Yes/No). Education level was dichotomized into attending or not attending college/university. Total household income was dichotomized into < or ≥ level of the lowest income quintile. Diabetes status was coded as ‘Diabetes mellitus’ (Yes/No). Use of hormonal contraceptives was determined from the question: ‘Do you currently use any prescription drug that influences the menstruation? Including oral or dermal contraceptives, intra uterine device with hormones or similar’ (Yes/No). Glycated haemoglobin (HbA1c,%) was measured from EDTA-blood samples and determined by high-performance liquid chromatography using an automated analyzer (Variant II, Bio-Rad Laboratories INC., Hercules, CA, USA). The total analytical coefficient of variation was <3.0%. This analysis has been certified by the National Glycohemoglobin Standardization Program (NGSP) as having documented traceability to the Diabetes Control and Complication Trial (DCCT) reference method [32]. Cut-off values for pre-diabetes (HbA1c 6.0–6.4%) and diabetes (HbA1c ≥6.5%) have been suggested by an international expert committee [33]. Missing data on any covariates led to the observations (*n* = 393) being excluded from the multivariable regression analysis.

### Statistical analysis

The interrelationships between BMI and WC and *S. aureus* nasal colonization were evaluated in logistic regression models stratified by sex.

As established thresholds for the associations between BMI, WC and *S. aureus* nasal colonization are lacking, BMI categories (<22.5, 22.5–<25.0, 25.0–<27.5, 27.5–<30.0, 30.0–<32.5, ≥32.5 kg/m^2^), WC quintiles among women (<80, 80–86, 87–92, 93–100, ≥101 cm) and WC quintiles among men (<91, 91–95, 96–101, 102–107, ≥108 cm) were defined. Selected characteristics of women and men were compared using age-adjusted regression analysis with linear *Ptrend* across all BMI categories (Table 1). Continuous BMI and WC data were used as predictors in the logistic regression models for the total population of women and men. As the analysis using the BMI categories (6 levels) and WC quintiles did not support a linear dose-response relationship with *S. aureus* nasal colonization, we chose not to use the continuous predictors in the analysis stratified by age groups.

**Table 1:** Characteristics of women and men by body mass index (BMI) categories.

	BMI (kg/m^2^)																										
	Total *n*		<22.5			22.5–<25.0			25.0–<27.5				27.5–<30.0				30.0–<32.5				≥32.5				*Ptrend* b		
**Women**																											
Numbers		2,169	442^a^			470^a^				462^a^				348^a^				223^a^				224^a^					
Age, years (SD)		2,169	51.9		(12.7)	52.1		(12.1)		55.5		(12.6)		57.2		(12.8)		57.5		(13.5)		54.7		(12.6)		<0.001	
Lower education (%)^c^		2,128	235		(54.2)	250		(53.3)		296		(65.9)		241		(71.3)		155		(70.5)		154		(70.6)		<0.001	
Low household income (%)^d^		1,970	80		(19.9)	86		(20.0)		107		(24.9)		96		(30.6)		50		(25.6)		69		(34.9)		<0.006	
Residing with children (%)		1,862	183		(46.8)	183		(45.2)		148		(37.3)		91		(31.3)		62		(33.0)		71		(37.4)		0.69	
Height, cm (SD)		2,169	165.1		(6.2)	164.6		(6.0)		163.2		(6.0)		163.0		(6.8)		163.5		(6.6)		163.0		(6.6)		<0.001	
Diabetes mellitus (%)		2,115	7		(1.6)	11		(2.4)		10		(2.2)		13		(3.9)		19		(8.8)		23		(10.7)		<0.001	
HbA1c,% (SD)		2,109	5.4		(0.4)	5.4		(0.4)		5.5		(0.5)		5.6		(0.6)		5.8		(0.7)		5.9		(0.9)		<0.001	
Recent Hospitalization (%)^e^		2,134	53		(12.2)	35		(7.5)		52		(11.4)		45		(13.2)		20		(9.2)		34		(15.5)		0.22	
Low physical activity (%)^f^		2,018	46		(11.2)	74		(16.8)		75		(17.3)		76		(23.8)		48		(23.5)		65		(31.7)		<0.001	
Current daily smoking (%)		2,135	123		(27.9)	108		(23.3)		95		(20.8)		73		(21.4)		35		(16.1)		32		(14.8)		<0.001	
Alcohol intake ≥2 times a week (%)		2,135	93		(21.3)	107		(23.0)		79		(17.3)		50		(14.6)		33		(15.4)		26		(11.9)		<0.001	
Atopic eczema (%)		1,912	38		(9.7)	39		(9.1)		47		(11.6)		22		(7.3)		19		(10.1)		22		(11.5)		0.34	
Psoriasis (%)		1,957	32		(7.9)	33		(7.7)		43		(10.4)		32		(10.1)		22		(11.0)		22		(9.4)		0.10	
**Men**																											
Numbers	1,709			132^a^		334^a^			479^a^				410^a^				214^a^				140^a^						
Age, years (SD)	1,709			53.7	(14.5)	52.9	(13.4)		54.0		(12.6)		53.6		(12.1)		53.6		(12.3)		54.4		(10.8)		0.46		
Lower education (%)^c^	1,696			76	(57.6)	177	(53.6)		282		(59.4)		251		(61.7)		140		(65.7)		88		(63.3)		0.01		
Low household income (%)^d^	1,654			27	(21.4)	55	(17.0)		69		(14.9)		53		(13.4)		33		(15.9)		21		(15.3)		0.19		
Residing with children (%)	1,506			51	(43.6)	138	(47.1)		183		(43.2)		150		(41.3)		74		(38.3)		35		(30.2)		0.10		
Height, cm (SD)	1,709			178.0	(7.6)	177.9	(6.8)		177.1		(6.9)		177.4		(6.8)		177.5		(7.1)		175.8		(7.1)		0.02		
Diabetes mellitus (%)	1,675			2	(1.5)	4	(1.2)		12		(2.5)		14		(3.5)		9		(4.4)		10		(7.4)		<0.001		
HbA1c,% (SD)	1,679			5.5	(0.4)	5.5	(0.5)		5.6		(0.7)		5.7		(0.5)		5.7		(0.6)		5.9		(0.8)		<0.001		
Recent Hospitalization (%)^e^	1,692			14	(10.6)	28	(8.5)		31		(6.5)		57		(14.0)		24		(11.3)		14		(10.3)		0.10		
Low physical activity (%)^f^	1,635			25	(20.2)	54	(16.8)		68		(14.8)		84		(21.5)		51		(25.0)		49		(36.3)		<0.001		
Current daily smoking (%)	1,695			45	(34.4)	64	(19.2)		91		(19.1)		63		(15.6)		36		(17.0)		29		(21.2)		0.01		
Alcohol intake ≥2 times a week (%)	1,690			38	(29.2)	93	(28.2)		115		(24.2)		94		(23.1)		36		(17.3)		21		(15.1)		<0.001		
Atopic eczema (%)	1,534			10	(8.6)	23	(7.5)		35		(8.0)		30		(8.2)		9		(5.0)		8		(6.25)		0.34		
Psoriasis (%)	1,558			10	(8.6)	30	(9.6)		27		(6.2)		39		(10.4)		18		(9.5)		14		(10.7)		0.37		

The Tromsø Staph and Skin Study (*n* = 3,878)^a^.

Values are given as means (standard deviation), and numbers (%).

*n*, numbers; SD, standard deviation; HbA1c, glycated haemoglobin.

a Numbers may vary due to missing information.

b *P* for trend, age-adjusted.

c < College/university degree.

d < Level of the lowest income quintile.

e Hospitalization last 12 months.

f Mostly sedentary recreational physical activity level like watching TV.

On the basis of biological plausibility and model fit, the variables age (continuous), DM, current daily smoking, education level and total household income were included as covariates in the multivariable regression model [13], [16], [29], [34], [35]. Chronic inflammatory skin diseases have been associated with *S. aureus* nasal colonization [12], [36]. However, self-reported atopic eczema and psoriasis were not associated with the main predictors (BMI and WC) and did not alter the estimated odds ratios (ORs) when included as covariates in the current analysis; thus skin diseases were omitted from the final model. We explored possible interactions with age in logistic regression models stratified by age tertiles (30–43, 44–59, and 60–87 years) among men and women and by proposed pre-/postmenopausal age ranges (30–54 and 55–87 years) among women. All the presented ORs and 95% Confidence Intervals (CIs) for *S. aureus* nasal colonization were generated from the multivariable logistic regression model described above, if not otherwise stated. To control for possible confounding by pre-diabetes and undiagnosed diabetes, sensitivity analysis restricted to those with HbA1c <6.0% (*n* = 3,207) was performed. Use of hormonal contraceptives has been found to increase the risk of *S. aureus* nasal carriage among young women [37]. In our study, 36% (238/667) of women aged 30–43 years, and 28% (298/1,083) of women 30–54 years reported current use of hormonal contraceptives. To control for possible confounding by these exogenous hormones, additional restriction analysis, including only non-users of hormonal contraceptives, was performed among young and premenopausal women.

Tests of model fit were performed by the Hosmer-Lemeshow goodness-of-fit test. Tests for linear trend were performed by assigning consecutive integers to each BMI and WC category, and testing whether the slope coefficient differed from zero using the Wald χ^2^ test. Tests for interaction between age strata and categories of BMI and WC (using age tertiles and pre-/postmenopausal age ranges as dummy variables) were done by inclusion of the multiplicative terms of the variables in the models, in both the total and the restricted sample. Tests of statistical significance were done by the likelihood ratio test comparing models with and without the multiplicative interaction terms. Two-sided *P*-values <0.05 were considered statistically significant. Statistical analyses were performed using STATA version 12.0 (StataCorp. 2011, *Stata Statistical Software: Release 12*, College Station, TX, USA).

## Results

The mean age of the 2,169 women in the study was 54.4 years and the mean BMI was 26.6 kg/m^2^, whereas the mean age of the 1,709 men was 53.6 years and the mean BMI was 27.3 kg/m^2^. In both sexes, high BMI was associated with DM, higher serum HbA1c level, sedentary leisure time activity, non-smoking, low alcohol intake and lower education level. Among women, high BMI was associated with increased age and lower total household income (all *Ptrend* <0.05, age-adjusted) (Table 1). The prevalence of self-reported atopic eczema and psoriasis were not associated with BMI in neither women nor men. The prevalence of *S. aureus* nasal colonization was 28.7% (1,113/3,878) and the sex-specific rates were 23.0% (498/2,169) among women, and 36.0% (615/1,709) among men, respectively.

### Odds of *S. aureus* nasal colonization by body mass index

**Figure 1:**
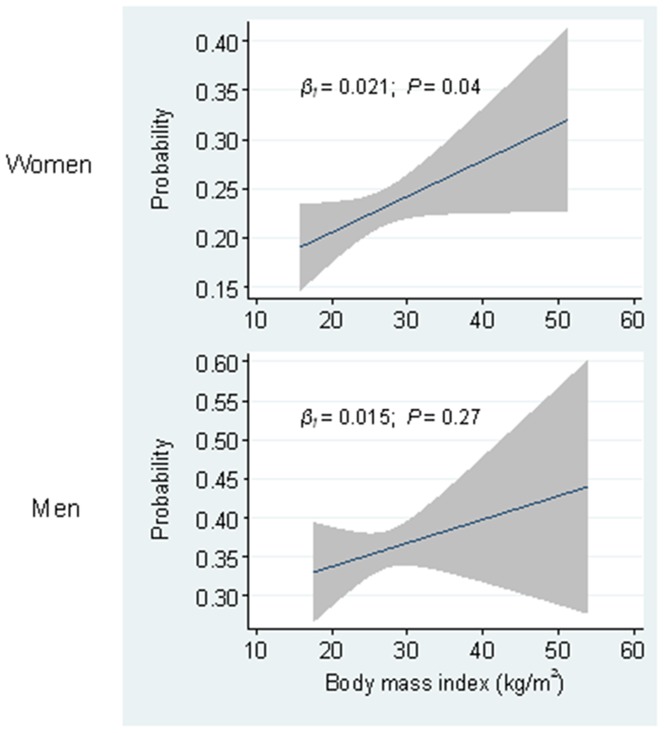
Probability of *Staphylococcus aureus* colonization. Probability of *S. aureus* carriage among women (*n* = 2,169) and men (*n* = 1,709) according to body mass index in kg/m^2^. Lines depict regression line (navy) with 95% mean prediction interval (grey area) from age-adjusted linear regression models.

There was a positive relationship between BMI (continuous) and *S. aureus* nasal colonization among women (Figure 1, age-adjusted). For each 2.5 kg/m^2^ increase in BMI a 7% increase in the odds of *S. aureus* nasal colonization was observed (multivariable model; OR 1.07, 95% CI 1.01–1.14). The prevalence of *S. aureus* nasal colonization increased from 20.6% (95% CI 16.8–24.4) among women with BMI <22.5 kg/m^2^ to 28.1% (95% CI 22.2–34.0) among women with BMI ≥32.5 kg/m^2^, corresponding to a 67% increased odds (BMI ≥32.5 versus <22.5 kg/m^2^) (Table 2). In sensitivity analysis restricted to women with HbA1c <6.0%, the OR was attenuated to 1.07 for each 2.5 kg/m^2^ increase in BMI (95% CI 1.00–1.14, *P* = 0.06) and 1.56 for BMI ≥32.5 kg/m^2^ versus <22.5 kg/m^2^ (95% CI 0.99–2.46, *P* = 0.06). BMI was not associated with *S. aureus* nasal colonization among men.

**Table 2:** Estimated odds ratios (ORs) for *S. aureus* nasal colonization by body mass index (BMI) categories among women and men.

BMI	Total	Colonized				
(kg/m^2^)	*n*	*n*	(%)	Crude OR	Crude OR^a^	OR^b^ (95% CI)
**Women (*n* = 2,169)**						
<22.5	442	91	(20.6)	ref	ref	ref
22.5–<25.0	470	113	(24.0)	1.22 (0.89–1.67)	1.22 (0.87–1.70)	1.20 (0.85–1.68)
25.0–<27.5	462	102	(22.1)	1.09 (0.79–1.50)	1.24 (0.88–1.73)	1.23 (0.87–1.73)
27.5–<30.0	348	76	(21.8)	1.08 (0.76–1.52)	1.11 (0.77–1.61)	1.10 (0.75–1.60)
30.0–<32.5	223	53	(23.8)	1.20 (0.82–1.77)	1.38 (0.92–2.09)	1.30 (0.85–1.98)
≥32.5	224	63	(28.1)	1.51 (1.04–2.19)	1.82 (1.22–2.72)	1.67 (1.11–2.52)
*Ptrend*				*0.11*	*0.01*	*0.04*
**Men (*n* = 1,709)**						
<22.5	132	46	(34.9)	ref	ref	ref
22.5–<25.0	334	118	(35.3)	1.02 (0.67–1.56)	0.93 (0.60–1.44)	0.86 (0.55–1.34)
25.0–<27.5	479	167	(34.9)	1.00 (0.67–1.50)	0.96 (0.63–1.45)	0.92 (0.61–1.41)
27.5–<30.0	410	153	(37.3)	1.11 (0.74–1.67)	1.03 (0.67–1.56)	0.98 (0.64–1.51)
30.0–<32.5	214	76	(35.5)	1.03 (0.65–1.62)	0.98 (0.61–1.56)	0.96 (0.60–1.54)
≥32.5	140	55	(39.3)	1.21 (0.74–1.98)	1.10 (0.66–1.82)	1.09 (0.65–1.83)
*Ptrend*				*0.40*	*0.54*	*0.45*

The Tromsø Staph and Skin Study (*n* = 3,878).

*n*, numbers; OR, odds ratios; CI, confidence interval.

a Unadjusted logistic regression model restricted to without missing data on any covariates (Women, *n* = 1,883; Men, *n* = 1,602).

b Multivariable logistic regression model including: age, current smoking (yes/no), diabetes mellitus (yes/no), education level (< or ≥ college/university degree), and household income (< or ≥ level of the lowest income quintile).

Among women aged 30–43 years, the odds of nasal colonization was 2.60 times higher for those with BMI ≥32.5 kg/m^2^ versus <22.5 kg/m^2^, whereas in older women no association was observed (*P* for interaction = 0.15 and 0.42) (Table 3). Among women aged 30–54 years, the odds of nasal colonization was 1.90 times higher for those with BMI ≥32.5 kg/m^2^ versus <22.5 kg/m^2^ (95% CI 1.10–3.29), whereas in older women no association was observed (*P* for interaction = 0.91). When restricting the analysis to those with HbA1c <6.0%, the estimated ORs for women 30–43 years (*P* for interaction = 0.03) (Table S1), and 30–54 years (*P* for interaction = 0.67), remained essentially unchanged. Among men, there was no difference in odds of *S. aureus* nasal colonization between categories of BMI in any age group.

**Table 3:** Estimated odds ratios (ORs) for *S. aureus* nasal colonization by body mass index (BMI) in age tertiles of women and men.

	Women (*n* = 2,169) a			Men (*n* = 1,709) a			
BMI	Total	Colonized		Total		Colonized	
(kg/m^2^)	n^a^	*n* a(%)	OR^b^ (95% CI)	n^a^		*n* a(%)	OR^b^ (95% CI)
**30–43 years**							
<22.5	169	35 (20.7)	ref	59		30 (55.9)	ref
22.5–<25.0	169	38 (22.5)	1.02 (0.59–1.76)	130		50 (38.5)	0.58 (0.31–1.09)
25.0–<27.5	130	33 (25.4)	1.31 (0.75–2.30)	152		61 (40.1)	0.68 (0.37–1.25)
27.5–<30.0	84	19 (22.6)	1.04 (0.54–2.03)	132		51 (38.6)	0.66 (0.35–1.25)
30.0–<32.5	54	14 (25.9)	1.26 (0.59–2.70)	73		27 (37.0)	0.60 (0.29–1.24)
≥32.5	71	26 (36.6)	2.60 (1.35–4.98)	36		16 (44.4)	0.85 (0.36–1.97)
*Ptrend*			*0.01*				*0.75*
**44–59 years**							
<22.5	148	29 (19.6)	ref		31	11 (35.5)	ref
22.5–<25.0	168	45 (26.8)	1.35 (0.76–2.38)		101	41 (40.6)	1.06 (0.45–2.51)
25.0–<27.5	149	29 (19.5)	1.11 (0.61–2.01)		183	68 (37.2)	1.04 (0.46–2.36)
27.5–<30.0	101	18 (17.8)	1.01 (0.51–2.01)		147	53 (36.1)	0.92 (0.40–2.11)
30.0–<32.5	68	18 (26.5)	1.63 (0.80–3.29)		63	25 (39.7)	1.21 (0.48–3.04)
≥32.5	57	11 (19.3)	1.06 (0.46–2.44)		63	26 (41.3)	1.06 (0.42–2.68)
*Ptrend*			*0.69*				*0.91*
**60–87 years**							
<22.5	125	27(21.6)	ref		145	32 (22.1)	ref^c^
22.5–<25.0	133	30 (22.6)	1.25 (0.63–2.47)				
25.0–<27.5	183	40 (21.9)	1.26 (0.67–2.37)		144	38 (26.4)	1.18 (0.65–2.13)
27.5–<30.0	163	39 (23.9)	1.14 (0.59–2.20)		131	49 (37.4)	1.90 (1.07–3.37)
30.0–<32.5	101	21 (20.8)	1.00 (0.46–2.14)		78	24 (30.8)	1.49 (0.77–2.86)
≥32.5	96	26 (27.1)	1.60 (0.77–3.32)		41	13 (31.7)	1.34 (0.58–3.12)
*Ptrend*			*0.50*				*0.14*

The Tromsø Staph and Skin Study (*n* = 3,878)^a^.

*n*, numbers; CI, confidence intervals.

*P* for interaction using BMI categories as *Ptrend* and age tertiles (middle versus lowest), among women: 0.15 and men: 0.66; age tertiles (highest versus lowest), among women: 0.42 and men: 0.11.

a Numbers may vary due to missing information.

b Multivariable logistic regression model including: current smoking (yes/no), diabetes mellitus (yes/no), education level (< or ≥ college/university degree), and household income (< or ≥ level of the lowest income quintile).

c Among men 60–87 years, the BMI categories <22.5 and 22.5–<25.0 kg/m^2^ were put together due to small numbers.

Among women aged 30–43 years with HbA1c <6.0%, further restriction to non–users of hormonal contraceptives gave a 2.85 times higher odds of nasal colonization for those with BMI ≥32.5 kg/m^2^ versus <22.5 kg/m^2^ (95% CI 1.21–6.74, *P* = 0.02), whereas among users of hormonal contraceptives there was no association (*P* for interaction = 0.58). Among premenopausal women aged 30–54 with HbA1c <6.0% and not using hormonal contraceptives the odds was attenuated (OR = 1.53, 95% CI 0.81–2.88, *P* = 0.19).

### Odds of *S. aureus* nasal colonization by waist circumference

Mean WCs were 91 cm and 100 cm among women and men, respectively. There was a positive relationship between WC (continuous) and *S. aureus* nasal colonization among women. For each 5-cm increase in WC a 6% increase in the odds of *S. aureus* nasal colonization was observed (multivariable model; OR 1.06, 95% CI 1.01–1.10). The prevalence of *S. aureus* nasal colonization among women increased from 19.8% (95% CI 15.8–23.8) in the 1st WC quintile to 25.3% (95% CI 21.2–29.4) in the 5th WC quintile corresponding to a 54% increased odds (WC ≥101 versus <80 cm) (Table 4). In sensitivity analysis restricted to women with HbA1c <6.0%, the OR was attenuated to 1.04 for each 5-cm increase in WC (95% CI 0.98–1.09, *P* = 0.16) and to 1.44 for the 5th versus 1st WC quintile (95% CI 0.96–2.16, *P = *0.08). In the male population, there was a U-shaped pattern in the prevalence of *S. aureus* nasal colonization by WC quintiles; 1st quintile (<91 cm), 39.0% (95% CI 33.6–44.5); 4th WC quintile (102–107 cm), 33.5% (95% CI, 28.5–38.6); and 5th quintile (≥108 cm), 38.8% (95% CI 33.6–44.0) (Table 4). No differences in odds of *S. aureus* nasal colonization were observed between men in different quintiles of WC using multivariable analysis.

**Table 4:** Estimated odds ratios (ORs) for *S. aureus* nasal colonization by waist circumference (WC) among women and men, The Tromsø Staph and Skin Study (*n* = 3,775).

WC-	Total	Colonized				
quintiles^a^	*n*	*n*	(%)	Crude OR	Crude OR^a^	OR^b^ (95% CI)
**Women (*n* = 2,115)**						
1st quintile	383	76	(19.8)	ref	ref	ref
2nd quntile	451	112	(24.8)	1.33 (0.96–1.86)	1.35 (0.94–1.93)	1.35 (0.94–1.93)
3rd quintile	398	81	(20.4)	1.03 (0.73–1.46)	1.06 (0.73–1.55)	1.08 (0.74–1.58)
4th quintile	449	105	(23.4)	1.23 (0.88–1.72)	1.36 (0.95–1.94)	1.36 (0.94–1.95)
5th quintile	434	110	(25.3)	1.37 (0.98–1.91)	1.61 (1.12–2.31)	1.54 (1.06–2.23)
*Ptrend*				*0.16*	*0.02*	*0.04*
**Men (*n* = 1,660)**						
1st quintile	310	121	(39.0)	ref	ref	ref
2nd quntile	299	102	(34.1)	1.27 (0.92–1.75)	1.26 (0.90–1.75)	0.86 (0.61–1.21)
3rd quintile	377	135	(35.8)	1.03 (0.74–1.43)	1.08 (0.77–1.51)	0.95 (0.69–1.31)
4th quintile	334	112	(33.5)	1.11 (0.81–1.51)	1.13 (0.82–1.55)	0.85 (0.60–1.19)
5th quintile	340	132	(38.8)	1.26 (0.92–1.72)	1.19 (0.85–1.65)	1.08 (0.77–1.51)
*Ptrend*				*0.93*	*0.63*	*0.70*

*n*, numbers; OR, odds ratios; CI, confidence interval.

a WC quintiles (cm); Women: 1st <80, 2nd 80–86, 3rd 87–92, 4th 93–100, 5th ≥101; Men: 1st <91, 2nd 91– 95, 3rd 96–101, 4th 102–107, 5th ≥108.

b Unadjusted logistic regression model restricted to those without missing data on any of the covariates (Women, *n* = 1,840, Men, *n* = 1,557).

b Multivariate logistic regression model including: age, current smoking (yes/no), diabetes mellitus (yes/no), education level (< or ≥ college/university degree), and household income (< or ≥ level of the lowest income quintile).

Among women aged 30–43 years, being in the 5th versus the 1st WC quintile was associated with a 2.12 times higher odds of *S. aureus* nasal colonization, whereas in older women no association was observed (*P* for interaction = 0.58 and 0.19) (Table 5). Among women aged 30–54 years, the corresponding odds of nasal colonization was 2.00 (95% CI 1.22–3.26), whereas in older women no association was observed (*P* for interaction = 0.44). Among men aged 30–43 years, being in the 5th and the 1st WC quintiles were associated with a 1.88 times higher odds of *S. aureus* nasal colonization, compared with being in the 4th WC quintile. Among men aged 60–87 years, being in the 2nd WC quintile was associated with a 2.62 times increased odds of *S. aureus* nasal colonization compared to being in the 1st WC quintiles (*P* for interaction = 0.69 and 0.32) (Table 5). When restricting the analysis of WC to those with HbA1c <6.0%, the estimated ORs among women and men aged 30–43 years, men aged 60–87 years (Table S2), and women aged 30–54 years (*P* for interaction = 0.21), remained essentially unchanged.

**Table 5:** Estimated odds ratios (ORs) for *S. aureus* nasal colonization by waist circumference (WC) in age tertiles of women and men, The Tromsø Staph and Skin Study (*n* = 3,775)^a^.

	Women (*n* = 2,115) a				Men (*n* = 1,660) a			
WC- quintiles^b^	Total	Colonized			Total		Colonized	
	*n* a	*n* a(%)	OR^c^ (95% CI)		*n* a		*n* a(%)	OR^c^ (95% CI)
**30–43 years**								
1s^t^ quintile	160	30 (18.8)	ref			139	67 (48.2)	1.88 (1.08–3.28)
2nd quntile	156	41 (26.3)	1.36 (0.78–2.39)			115	38 (33.0)	1.08 (0.60–1.94)
3rd quintile	125	27 (21.6)	1.13 (0.62–2.05)			123	54 (43.9)	1.81 (1.03–3.21)
4th quintile	115	29 (25.2)	1.45 (0.80–2.64)			99	31 (31.1)	ref
5th quintile	108	35 (32.1)	2.12 (1.17–3.85)			84	38 (45.2)	1.88 (1.01–3.49)
*Ptrend*			*0.03*					*0.68*
**44–59 years**								
1st quintile	119	21 (17.7)	ref			92	38 (41.3)	ref
2nd quntile	156	39 (25.0)	1.93 (0.99–3.74)			106	37 (34.9)	0.76 (0.42–1.38)
3rd quintile	122	22 (18.0)	1.35 (0.64–2.82)			134	52 (38.8)	0.89 (0.51–1.56)
4th quintile	152	33 (21.7)	1.74 (0.88–3.44)			119	45 (37.8)	0.95 (0.53–1.69)
5th quintile	124	29 (23.4)	2.03 (1.00–4.11)			120	47 (39.2)	0.90 (0.50–1.62)
*Ptrend*			*0.15*					*0.95*
**60–87 years**								
1st quintile	104	25 (24.0)	ref			79	16 (20.3)	ref
2nd quntile	139	32 (23.0)	0.85 (0.43–1.71)			78	27 (34.6)	2.62 (1.18–5.80)
3rd quintile	151	32 (21.2)	0.76 (0.38–1.50)			120	29 (24.2)	1.31 (0.61–2.80)
4th quintile	182	43 (23.6)	0.88 (0.46–1.68)			116	36 (31.0)	1.65 (0.78–3.49)
5th quintile	202	46 (22.8)	0.88 (0.45–1.69)			136	47 (34.6)	1.93 (0.94–3.98)
*Ptrend*			*0.85*					*0.34*

*n*, numbers; CI, confidence interval.

P for interaction using WC quintiles as *Ptrend* and age tertiles (middle versus lowest), among women: 0.58 and men: 0.69; age tertiles (highest versus lowest), among women: 0.19 and men: 0.32.

a Numbers may vary due to missing information.

b WC quintiles (cm); Women: 1st <80, 2nd 80–86, 3rd 87–92, 4th 93–100, 5th ≥101; Men: 1st <91, 2nd 91– 95, 3rd 96–101, 4th 102–107, 5th ≥108.

c Multivariable logistic regression model including: current smoking (yes/no), diabetes mellitus (yes/no), education level (< or ≥ college/university degree), and household income (< or ≥ level of the lowest income quintile).

Furthermore, when restricting the analyses of young women aged 30–43 years, with HbA1c <6.0%, to non-users of hormonal contraceptives, being in the 5th versus the1st WC quintiles was associated with a 2.36 (95% CI 1.09–5.08; *P* = 0.03) times increased odds of *S. aureus* nasal colonization, whereas, in the same restriction analysis among premenopausal women aged 30–54 years, a 2.04 times increased odds was observed (95% CI 1.10–3.78; *P* = 0.02). Among young and premenopausal women with HbA1c <6.0% who were hormonal contraceptives users, there was no association between WC and *S. aureus* nasal colonization (*P* for interaction = 0.52 for age 30–43 years and 0.76 for age 30–54 years).

## Discussion

To our knowledge, this is the first report to show that women with higher BMI and WC have increased odds of *S. aureus* nasal colonization independent of pre-diabetes and diabetes, suggesting that excess body weight may be a marker of increased susceptibility to colonization. The association seemed to be restricted to young and premenopausal women. The current study indicates that a threshold effect of fat mass may be more important than a dose-response effect on *S. aureus* nasal colonization. Among women aged 30–43 years, being obese as compared with being lean was associated with a 2.6 times increased odds of *S. aureus* nasal colonization, and having a WC >101 cm versus <80 cm was associated with a 2.1 times increased odds of colonization.

The observed association between high BMI and increased odds of *S. aureus* nasal colonization in our study is in line with findings in the general US population >19 years examined in 2001–04; NHANES reported a 1.3 and 1.2 times increased odds of *S. aureus* nasal colonization among obese (BMI ≥30 kg/m^2^) men and women, respectively, compared with normal or overweight subjects. The associations in NHANES were independent of age group, race, ethnicity, and survey cycle, whereas diabetes was omitted from the final models because it was not significantly associated with the outcome [13]. Our results are also supported by a study among 4,030 adult surgical patients observing that obesity was an independent risk factor for preoperative *S. aureus* nasal colonization when adjusting for age, sex, current smoking and previous use of antimicrobial agents [14].

High serum glucose and DM have been associated with increased risk of *S. aureus* nasal colonization, and it has been suggested that high levels of blood or mucosal glucose may influence phagocyte function, bacterial adherence and colonization [16]–[18]. Importantly, the current study shows associations between BMI and WC and *S. aureus* nasal colonization independent of pre-diabetes or diabetes, and thus extends previous findings. The reasons for these associations are unclear, but may include physical, biochemical, or hormonal factors.

Studies in humans and animals have suggested that adiposity in itself may cause impaired immune responses through immunomodulatory effects of changes in reproductive hormones [19]–[23], and that obesity may cause a chronic low-grade inflammation associated with an impaired immune response [2], [25].

We identified BMI and WC as significant predictors of *S. aureus* nasal colonization among younger and premenopausal women and that the associations remained unchanged among non-users of hormonal contraceptives, whereas was no association among older and postmenopausal women. Thus, we hypothesize that possible effect modification by age group and menopausal status may be linked to levels of sex hormones. Among premenopausal women obesity has been linked to anovulatory cycles with lower circulating estrogen levels and higher androgen levels [22], [38], while in obese postmenopausal women estrogen levels are increased [20]. Androgens generally exert suppressive effects on both innate and adaptive immune responses [21]. Estrogens on the other hand, exert immune enhancing activities, a mechanism that may have evolved to protect females from infection [19], [21]. Interestingly, the expression of antimicrobial peptides (AMPs), some of which are associated with *S. aureus* skin infections and nasal colonization [39], [40], are modified by sex hormones in other body sites, i.e. the genital tract [41]–[43].

In this study, we observed that the association between BMI and *S. aureus* nasal colonization was modified by sex. This is in contrast to others who have observed increased odds of colonization among both obese women and men [13]. Nevertheless, the current study also suggests a U-shaped relationship between WC and *S. aureus* nasal colonization among young men. This may reflect non-causal associations or sex-associated differences in lean and fat body mass. Studies have identified higher *S. aureus* nasal colonization rates among men [7], [13], [14] and previous reports have shown that predictors of *S. aureus* nasal colonization may vary by sex [7], [13]. Obesity in men has been associated with higher levels of estrogens and lower levels of androgens [23], opposite to the changes in sex hormone levels seen among young women. Furthermore, obesity may induce a low-grade chronic inflammation that attenuates leptin signalling. It has been hypothesized that leptin signalling may be important for immune function, including cutaneous antimicrobial defense [2], [44]. Leptin is an adipokine predominately expressed in subcutaneous adipocytes [45], and one may speculate that gender differences in subcutaneous fat accumulation [46], leptin resistance [47] and sex hormone levels [20], [22], [23] may contribute to the observed differences in obesity-related susceptibility to colonization between women and men.

This large population-based study was subject to limitations. The cross-sectional study design is not capable of establishing or refuting a causal relationship between obesity and *S. aureus* nasal colonization. Thus, future prospective studies of long term effects of obesity and weight change on the risk of *S. aureus* colonization and subsequent infections are needed. Even though multiple testing was performed in the current analysis, the primary hypothesis was tested in all the statistical models, thus reducing the risk of chance findings. Furthermore, subgroup analysis was done as a result of formal tests of interaction. Although we adjusted our analysis for important risk factors of nasal colonization, uncontrolled or residual confounding might have influenced the results. Missing data on skin infections in the TSSS is a potential source of residual confounding [12], [36]. Importantly, we performed sensitivity analysis to minimize the effect of confounding by diabetes and use of hormonal contraceptives.

In this study with a cross-sectional design, we know the colonization state only at the time of the study. However, based on the results from a large substudy of 2,868 individuals with a second nasal swab culture in the TSSS, we may assume that the misclassification rate of non-colonized as colonized was low. In this substudy, 113 (3.9%) were misclassified as colonized from the culturing results of the first nasal swab. The misclassification is yet, non-differential as it was independent of the main predictors, which may have biased our OR estimates towards one.

The nares were the only body sites sampled, whereas colonization may occur also in other sites such as the throat, axillae, perineum, and skin [8], [48]–[50]. However, as decolonization of the nose usually has a decolonizing effect on the skin, the nose is assumed to be the major site of *S. aureus* colonization [51]. Furthermore, nasal colonization seems to play a key role in the epidemiology and pathogenesis of staphylococcal infection [8]–[11].

In conclusion, our study indicates positive associations of general and abdominal obesity and *S. aureus* nasal colonization among younger and premenopausal women independent of pre-diabetes and DM. High WC may also be a risk factor for *S. aureus* nasal colonization among young men. Given causality can be established, the suggested effects on *S. aureus* colonization in our study may offer new and important perspectives for the prevention of *S. aureus* disease in the population. However, the role of body weight and adiposity at different ages and by sex should be addressed in future prospective studies to improve our ability to identify high risk groups and to target effective prevention of *S. aureus* colonization and disease.

## Supporting Information

Table S1 **Estimated odds ratios (ORs) for** ***S. aureus*** **nasal colonization by body mass index (BMI) in age tertiles of women and men with HbA1c <6.0%.** The Tromsø Staph and Skin Study (*n* = 3,207)^a^.
(DOCX)

Table S2 **Estimated odds ratios (ORs) for** ***S. aureus*** **nasal colonization by waist circumference (WC) in age tertiles of women and men with HbA1c <6.0%.** The Tromsø Staph and Skin Study (*n* = 3,129)^a^.
(DOCX)
