# Supplementary material for: Obesity and Staphylococcus aureus Nasal Colonization among Women and Men in a General Population
Source: PLoS One. 2013 May 7;8(5):e63716. doi: 10.1371/journal.pone.0063716 (PMC3646820; doi:10.1371/journal.pone.0063716)
Supplement: Table S1 — Estimated odds ratios (ORs) for S. aureus nasal colonization by body mass index (BMI) in age tertiles of women and men with HbA1c <6.0%. The Tromsø Staph and Skin Study (n = 3,207)a. (DOCX) [file pone.0063716.s001.docx]

| **Table S1.** Estimated odds ratios (ORs) for *S. aureus* nasal colonization by body mass index (BMI) in age tertiles of women and men with HbA1c <6.0%. The Tromsø Staph and Skin Study (*n* = 3,207)^a^. | | | | | | | |
| --- | --- | --- | --- | --- | --- | --- | --- |
|  | **Women (*n* = 1,814)^a^** | | |  | **Men (*n* = 1,393)^a^** | | |
| **BMI** | **Total** | **Colonized** |  |  | **Total** | **Colonized** |  |
| **(kg/m^2^)** | ***n*^a^** | ***n*^a^(%)** | **OR^b^ (95% CI)** |  | ***n*^a^** | ***n*^a^(%)** | **OR^b^ (95% CI)** |
| **30–43 years** | | | | | | | |
| <22.5 | 166 | 34 (20.5) | ref |  | 56 | 29 (51.8) | ref |
| 22.5–<25.0 | 161 | 36 (22.4) | 1.02 (0.59–1.78) |  | 122 | 46 (37.7) | 0.53 (0.27–1.02) |
| 25.0–<27.5 | 126 | 33 (26.2) | 1.39 (0.79–2.45) |  | 140 | 56 (40.0) | 0.64 (0.34–1.21) |
| 27.5–<30.0 | 77 | 17 (22.1) | 1.02 (0.51–2.04) |  | 119 | 48 (40.3) | 0.65 (0.34–1.26) |
| 30.0–<32.5 | 45 | 11 (24.4) | 1.25 (0.56–2.83) |  | 66 | 25 (37.9) | 0.61 (0.29–1.28) |
| ≥32.5 | 63 | 23 (36.5) | 2.56 (1.29–5.07) |  | 29 | 12 (41.4) | 0.69 (0.43–1.10) |
| *Ptrend* |  |  | *0.02* |  |  |  | *0.68* |
| **44–59 years** | | | | | | | |
| <22.5 | 134 | 28 (20.9) | ref |  | 28 | 10 (35.7) | ref |
| 22.5–<25.0 | 151 | 42 (27.8) | 1.29 (0.71–2.32) |  | 89 | 33 (37.1) | 0.90 (0.36–2.24) |
| 25.0–<27.5 | 130 | 25 (19.2) | 1.04 (0.56–1.95) |  | 162 | 63 (38.9) | 1.11 (0.47–2.63) |
| 27.5–<30.0 | 85 | 14 (16.5) | 0.82 (0.39–1.74) |  | 124 | 46 (37.1) | 0.97 (0.40–2.35) |
| 30.0–<32.5 | 58 | 13 (22.4) | 1.19 (0.55–2.56) |  | 45 | 19 (42.2) | 1.21 (0.44–3.30) |
| ≥32.5 | 42 | 6 (14.3) | 0.65 (0.23–1.86) |  | 34 | 12 (35.3) | 0.71 (0.23–2.16) |
| *Ptrend* |  |  | *0.46* |  |  |  | *0.89* |
| **60–87 years** | | | | | | | |
| <22.5 | 98 | 22 (22.5) | ref |  | 112^b^ | 21 (18.8)^b^ | ref^c^ |
| 22.5–<25.0 | 106 | 22 (20.8) | 1.01 (0.47–2.18) |  |  |  |  |
| 25.0–<27.5 | 149 | 30 (20.1) | 1.00 (0.50–2.02) |  | 99 | 29 (29.3) | 1.67 (0.83–3.37) |
| 27.5–<30.0 | 112 | 26 (23.2) | 0.95 (0.45–2.01) |  | 93 | 32 (34.4) | 1.89 (0.94–3.79) |
| 30.0–<32.5 | 55 | 10 (18.2) | 0.83 (0.32–2.19) |  | 56 | 16 (28.6) | 1.45 (0.66–3.20) |
| ≥32.5 | 56 | 14 (25.0) | 1.38 (0.59–2.24) |  | 19 | 7 (36.8) | 1.94 (0.62–6.03) |
| *Ptrend* |  |  | *0.72* |  |  |  | *0.20* |
| Abbreviations: *n*, numbers; CI, confidence intervals; odds ratio (OR).  *P* for interaction using BMI categories as *Ptrend* and age tertiles (middle versus lowest)*,* among women: 0.03 and men: 0.93; age tertiles (highest versus lowest), among women: 0.26 and men: 0.12.  ^a^ Numbers may vary due to missing information.  ^b^ Multivariable logistic regression model including: current daily smoking (yes/no), diabetes mellitus (yes/no), education level (< or ≥ college/university degree), and household income (< or ≥ level of the lowest income quintile).  ^c^ Among men 60–87 years, the BMI categories <22.5 and 22.5–<25.0 kg/m^2^ were put together due to small numbers. | | | | | | | |
