# Supplementary material for: Obesity and Staphylococcus aureus Nasal Colonization among Women and Men in a General Population
Source: PLoS One. 2013 May 7;8(5):e63716. doi: 10.1371/journal.pone.0063716 (PMC3646820; doi:10.1371/journal.pone.0063716)
Supplement: Table S2 — Estimated odds ratios (ORs) for S. aureus nasal colonization by waist circumference (WC) in age tertiles of women and men with HbA1c <6.0%. The Tromsø Staph and Skin Study (n = 3,129)a. (DOCX) [file pone.0063716.s002.docx]

| **Table S2.** Estimated odds ratios (ORs) for *S. aureus* nasal colonization by waist circumference (WC) in age tertiles of women and men with HbA1c <6.0%. The Tromsø Staph and Skin Study (*n* = 3,129)^a^ | | | | | | | | |
| --- | --- | --- | --- | --- | --- | --- | --- | --- |
|  | **Women (*n* = 1,772)^a^** | | |  | **Men (*n* = 1,357)^a^** | | | |
| **WC** | **Total** | **Colonized** |  |  | **Total** | **Colonized** | |  |
| **quintiles^b^** | ***n*^a^** | ***n*^a^(%)** | **OR^c^ (95% CI)** |  | ***n*^a^** | ***n*^a^(%)** | | **OR^c^ (95% CI)** |
| **30–44 years** | | | | | | | | |
| 1st quintile | 155 | 29 (18.7) | ref |  | 129 | 62 (48.1) | | 1.86 (1.04–3.30) |
| 2nd quntile | 154 | 41 (26.6) | 1.39 (0.79–2.45) |  | 111 | 38 (34.2) | | 1.13 (0.62–2.06) |
| 3rd quintile | 117 | 25 (21.4) | 1.11 (0.60–2.06) |  | 114 | 50 (43.9) | | 1.74 (0.97–3.14) |
| 4th quintile | 105 | 27 (25.7) | 1.50 (0.81–2.78) |  | 90 | 28 (31.1) | | ref |
| 5th quintile | 95 | 30 (31.6) | 2.06 (1.11–3.85) |  | 71 | 33 (46.5) | | 1.90 (0.98–3.66) |
| *Ptrend* |  |  | *0.04* |  |  |  | | *0.67* |
| **44–60 years** | | | | | | | | |
| 1st quintile | 113 | 21 (18.6) | ref |  | 85 | 35 (41.2) | ref | |
| 2nd quntile | 138 | 36 (26.1) | 1.98 (1.01–3.90) |  | 93 | 30 (32.3) | 0.71 (0.37–1.34) | |
| 3rd quintile | 110 | 19 (17.3) | 1.24 (0.58–2.65) |  | 116 | 48 (41.4) | 1.00 (0.56–1.81) | |
| 4th quintile | 130 | 28 (21.5) | 1.57 (0.78–2.19) |  | 97 | 37 (38.1) | 0.94 (0.51–1.75) | |
| 5th quintile | 92 | 18 (19.6) | 1.48 (0.68–3.21) |  | 79 | 30 (38.0) | 0.84 (0.44–1.63) | |
| *Ptrend* |  |  | *0.66* |  |  |  | *0.98* | |
| **60–87 years** | | | | | | | | |
| 1st quintile | 84 | 21 (25.0) | ref |  | 62 | 11 (17.7) | ref | |
| 2nd quntile | 115 | 25 (21.7) | 0.78 (0.37–1.67) |  | 63 | 22 (34.9) | 3.08 (1.23–7.73) | |
| 3rd quintile | 113 | 26 (23.0) | 0.74 (0.35–1.55) |  | 83 | 18 (21.7) | 1.26 (0.50–3.16) | |
| 4th quintile | 130 | 25 (19.2) | 0.62 (0.29–1.32) |  | 82 | 25 (30.5) | 1.85 (0.76–4.49) | |
| 5th quintile | 121 | 25 (20.7) | 0.78 (0.36–1.66) |  | 82 | 29 (35.4) | 2.19 (0.91–5.27) | |
| *Ptrend* |  |  | *0.43* |  |  |  | *0.36* | |
| *n*, numbers; OR, odds ratios; CI, confidence interval.  *P* for interaction using WC quintiles as a *Ptrend* and age tertiles (middle versus lowest), among women: 0.25 and men: 0.82; age tertiles (highest versus lowest), among women: 0.07 and men: 0.29.  ^a^ Numbers may vary due to missing information.  ^b^ WC quintiles (cm); Women: 1st <80, 2nd 80–86, 3rd 87–92, 4th 93–100, 5th ≥101; Men: 1st <91, 2nd 91–95, 3rd 96–101, 4th 102–107, 5th ≥108.  ^c^ Multivariable logistic regression model including: current daily smoking (yes/no), diabetes mellitus (yes/no), education level (< or ≥ college/university degree), and household income (< or ≥ level of the lowest income quintile). | | | | | | | | |
